# Factors Impacting Chinese Older Adults’ Intention to Prevent COVID-19 in the Post–COVID-19 Pandemic Era: Survey Study

**DOI:** 10.2196/53608

**Published:** 2024-04-17

**Authors:** Huixin Guan, Wei Wang

**Affiliations:** 1 USC-SJTU Institute of Cultural and Creative Industry Shanghai Jiao Tong University Shanghai China

**Keywords:** COVID-19, SARS-CoV-2, health protection, social capital, media exposure, negative emotions, structural influence model of communication, SIM, protect, protection, protective, intent, intention, prevention, preventative, restriction, restrictions, public health measures, safety, news, newspaper, media, radio, health communication, influence, influencing, infectious, infection control, pandemic, gerontology, geriatric, geriatrics, older adult, older adults, older person, older people, aging

## Abstract

**Background:**

Understanding the factors influencing individuals’ health decisions is a dynamic research question. Particularly, after China announced the deregulation of the COVID-19 epidemic, health risks escalated rapidly. The convergence of “no longer controlled” viruses and the infodemic has created a distinctive social period during which multiple factors may have influenced people’s decision-making. Among these factors, the precautionary intentions of older individuals, as a susceptible health group, deserve special attention.

**Objective:**

This study aims to examine the intention of older adults to engage in preventive behaviors and the influencing factors, including social, media, and individual factors, within the context of the postepidemic era. Drawing upon the structural influence model of communication, this study tests the potential mediating roles of 3 different types of media exposure between cognitive and structural social capital and protective behavior intention, as well as the moderating role of negative emotions between social capital and media exposure.

**Methods:**

In this study, a web survey was used to collect self-reported quantitative data on social capital, media exposure, negative emotions, and the intention to prevent COVID-19 among older adults aged ≥60 years (N=399) in China.

**Results:**

The results indicate that cognitive social capital significantly influenced protective behavior intention (*P*<.001), with cell phone exposure playing an additional impactful role (*P*<.001). By contrast, newspaper and radio exposure and television exposure mediated the influence of structural social capital on protective behavior intention (*P*<.001). Furthermore, negative emotions played a moderating role in the relationship between cognitive social capital and cell phone exposure (*P*<.001).

**Conclusions:**

This study suggests that using tailored communication strategies across various media channels can effectively raise health awareness among older adults dealing with major pandemics in China, considering their diverse social capital characteristics and emotional states.

## Introduction

### Background

In December 2022, the Chinese State Council issued the Notice on Further Optimizing the Implementation of COVID-19 Prevention and Control Measures, announcing the liberalization of COVID-19 control in China [1]. Following the policy change, there was a rapid and widespread surge in virus transmission. During this period, the health status of older adults became a matter of concern. Older adults, characterized by degenerating body functions, weakened immune systems, and underlying diseases [2], are generally at a disadvantage in fighting viral infections. Survey data showed that the mortality rate of COVID-19 is significantly higher among older adults aged ≥65 years than among younger adults globally. In China, the COVID-19 mortality rate is 2.3%, with >50% of deaths occurring in patients aged ≥50 years [3]. Thus, older adults are considerably more susceptible to the detrimental effects of COVID-19 than the general population. In the postepidemic era, it is crucial to examine the behavioral intentions of older adults to cope with disease risk.

This study aimed to address this research problem by introducing the structural influence model of communication (SIM), a research framework proven to be reliable in the context of large-scale health risks. In addition, this study aimed to enhance the SIM by delving into specific variables in depth, seeking to establish a more comprehensive research perspective on investigating influencing factors. This approach further examined the applicability of the model across a broader range of research contexts.

### Theory Framework

The SIM suggests that health cognition and behavior should be considered within the elements of the social system based on the social-ecological theory [4]. It builds on the basic idea that communication is controlled by power dynamics, similar to the knowledge gap theory, which suggests that individuals with lower socioeconomic status have limited access to information [5]. Accordingly, the model states that social determinants and the conditions of mobilizable resources greatly shape the information environment, leading to communication inequalities and then differentiated health behaviors and outcomes. However, most previous studies have focused on individual-level factors, such as age [6], education [7], and revenue [8,9], with minimal attention paid to the social resources that individuals may have access to [10]. In fact, a weak ability to mobilize social resources, that is, social capital, could often lead to a disadvantage in health information seeking [11,12] and health behaviors [13].

Furthermore, the communication inequality could manifest in multiple aspects, such as health message search frequency [14], message type [15,16], and media exposure preferences [17]. As media technology advances, disparities in the use of and exposure to traditional and new media, on the one hand, have become visible, resulting in suboptimal health behaviors among certain populations. Several studies support this perspective. For instance, Viswanath and Ackerson [13] explored the phenomenon of mass media exposure in the United States and concluded that social structures, such as race and class, lead to widespread health disparities.

Research on the SIM is an ongoing endeavor. Some scholars believe that more evidence is still needed to establish the relationship among social capital, communication, and health behavior [12,18]. One of the reasons is that current research based on the SIM does not clearly reveal the intrapersonal processes that guide communication decisions [11,19], whereas some other theories state that affective responses to risk, such as anxiety and fear, possibly relate to information seeking [20]. Hovick et al [11] argued that personal perception factors, particularly in the face of risk, can further influence communication inequalities and should be considered. In particular, risk perception can influence information-seeking attitudes and outcomes by shaping affective responses [20]. When individuals perceive threats as serious, it triggers negative emotions, such as fear and anxiety, motivating them to seek and process information to mitigate those emotions [21]. Individuals with a strong sense of risk and self-efficacy are more likely to engage in protective behaviors driven by negative emotions [22]. In this paper, we continue this line of thought and introduce the variable of negative emotions to expand the existing relationship among social capital, media exposure, and protective behavior intention, ultimately forming the theoretical framework.

### Variables and Hypotheses

#### Social Capital

Social capital is defined as a general public resource [23] that is typically found in relationships within kinship, professional, organizational, and neighborhood contexts [24]. Putnam [25,26] developed 2 classification schemes for social capital. The first scheme distinguishes between bonding and bridging components based on the inward and outward social connections of the group. The second scheme divides social capital into cognitive and structural aspects, with the cognitive element encompassing trust and reciprocity, whereas the structural element pertains to social participation and social networks [27].

Social capital has long been recognized as an influential factor in health [28], dating back to the 19th century [29], when Durkheim linked social integration to suicide rates [30]. Many studies have demonstrated a strong correlation between social capital and disease prevalence [31-34] as well as various types of health behavioral habits and lifestyles [35,36]. The SIM further emphasizes the significant role of social capital in the potential relationship among social structural factors, communication inequalities, and health inequalities [11,12].

Different types of social capital may play distinct roles in health outcomes. For example, bridging social capital is believed to provide individuals with new information and resources, enhancing their ability to address health problems [37]. Conversely, bonding social capital may restrict individuals’ access to information, increase psychological stress, and negatively impact health [38]. However, the differential effects of cognitive and structural social capital on communication and health outcomes have not received adequate attention. Kawachi and Berkman [39] identified two mechanisms through which social capital influences health: (1) social networks serve as information channels, facilitating the transfer and exchange of information among members, and (2) trust within the community accelerates the transmission of health behaviors. Building on this perspective, cognitive and structural elements operate through different pathways. Several empirical studies have concluded that cognitive social capital is consistently beneficial for mental health outcomes, whereas the outcomes associated with structural social capital are ambiguous [40,41]. Therefore, this study proposes the following hypothesis: cognitive (hypothesis 1a) and structural (hypothesis 1b) social capital positively predicts the intention to adopt protective behavior.

#### Media Exposure

The SIM encompasses a broad definition of communication [12], with most studies focusing on information seeking and information scanning [42,43] but paying less attention to preferences for accessing information from different types of media channels. This study specifically examined mass media exposure. It is important to note that social structural differences significantly influence the patterns of media exposure among specific populations. For instance, a study on women’s exposure to family planning messages in Nigeria revealed notable disparities between urban and rural women in terms of the likelihood of accessing information through media channels such as print media, radio, and television [44]. This finding underscores the significance of media exposure as an expression of communication inequality in the SIM. Moreover, numerous empirical studies have shown that different forms of media exposure can predict health prevention beliefs and behaviors. For example, Bleakley et al [45] showed that exposure to sexual content in media, including movies, television shows, music, magazines, and video games, influenced adolescents’ beliefs and subsequent behaviors. Sitar-Taut and Mican [46] identified social media exposure as the most expressive driver of attitudes toward vaccination.

Regarding older adults, there is a general belief that they prefer to seek health information from traditional media, such as newspapers and radio [47]. However, with the proliferation of media technologies, internet use among older adults has been on the rise [48]. New media platforms are increasingly playing a vital role in health interventions for older adults. A study conducted in Hong Kong [49] investigated the internet use of older adults and found that the use of media devices, such as phones and computers, can effectively improve the mental health of older adults. Another study by Yi [50] compared the use of paper-based media with the use of electronic media among older adults and discovered that both significantly promote healthy lifestyles. However, the mechanism through which paper-based media use influences older adults is associated with selective socioeconomic characteristics, whereas the effect of electronic media use plays a more direct role. Accordingly, this study proposes the following hypothesis: newspaper and radio exposure (hypothesis 2a), television exposure (hypothesis 2b), and cell phone exposure (hypothesis 2c) mediate the effects of cognitive social capital on protective behavior intention, and newspaper and radio exposure (hypothesis 2d), television exposure (hypothesis 2e), and cell phone exposure (hypothesis 2f) mediate the effects of structural social capital on protective behavior intention.

#### Negative Emotions

Negative emotions are emotional responses that arise in the face of health risks [51,52]. A strong emotional response can drive individuals to adopt adaptive behaviors and, ultimately, facilitate the reception of information. Although fear has often been considered a mediating factor in the process from risk perception to behavior adoption, it is important to note that fear is not the sole emotion triggered when individuals encounter threats. Other emotions such as worry, anger, and guilt also come into play [21,53]. Although some studies have indicated that both positive and negative emotional reactions affect people’s health behaviors [54,55], most studies focus on negative emotions and highlight their importance as predictors of individuals’ health behaviors among various factors [56].

According to the planned risk information-seeking model, negative emotions can underscore the need for risk information and initiate information-seeking behaviors [20]. When individuals experience feelings of worry, anger, or fear, they are motivated to actively search for relevant risk information to regain a sense of control over the situation [52]. The selective exposure theory suggests that emotional states prompt individuals to actively choose and use media that align with their emotional needs, with stressed people tending to opt for calming media and bored people more inclined to select uplifting media [57]. The study by Huang and Yang [58] of the Chinese public’s perception of air pollution noted that negative effects positively influenced people’s information-seeking behavior regarding air pollution. The appraisal theory of emotion posits that emotions stem from automatic and subjective evaluations of events [59]. Individuals’ assessments of their surroundings give rise to various emotional states [60]. For instance, those experiencing fear or anger are more inclined to seek information aligned with the action tendencies of their respective emotions while being less likely to pursue information incongruent with their current emotional state [61]. Given this framework, negative emotions, prevalent among individuals confronting health risks, are likely to exert a moderating influence on subsequent information-seeking and health behaviors. Furthermore, focusing on different media channels, negative emotions have been shown to increase individuals’ intention to seek health information from various sources, such as interpersonal, traditional, and web sources [54]. Consequently, this study proposes the following hypothesis: negative emotions play a moderating role between social capital and media exposure (hypothesis 3).

This study aimed to shed light on the influences of multiple social, personal, and interpersonal elements on individual health issues within the context of a larger social-ecological system. By integrating the SIM and planned risk information-seeking model, this study investigated the impact of social capital, media exposure, and negative emotions on the health promotion intention of older adults, a group that faces health disadvantages, in a postepidemic era. Specifically, this study measured the association between older adults’ cognitive and structural social capital and their intention to protect themselves from COVID-19 and highlighted the role of media exposure in this process of influence. Furthermore, the variable of negative emotions was also introduced to assess whether it moderates the relationship between social capital and media exposure.

## Methods

### Design and Participants

This study collected data in March 2023. We developed a web-based questionnaire through Wenjuan.com (Zhongyan Technology), a Chinese platform akin to Google Forms (Google LLC). Snowball sampling method was used in this study. We disseminated the web-based survey link on the WeChat (Tencent Inc) platform and encouraged our friends to share it with their older family members. The participants were limited to individuals aged ≥60 years. They were requested to complete the questionnaire, either in person or with the assistance of their family members. Each respondent was rewarded with a monetary incentive (RMB ¥5 [US $0.7]) after completing the survey.

Before the formal distribution of the questionnaire, a small-scale pretest was conducted over the web, and 46 completed questionnaires were collected. On the basis of the feedback received from the respondents, certain questions were modified. The final version of the questionnaire was then distributed on a large scale in March 2023, and 514 completed questionnaires were collected. However, 115 (22.4%) questionnaires were deemed invalid owing to incomplete answers or a completion time of <150 seconds. Therefore, a total of 399 valid questionnaires were included in the analysis, representing a completion rate of 77.6%. The age of the participants varied from 60 to 93 years, with an average age of 64.93 (SD 4.91) years. Table 1 presents the characteristics of the respondents.

**Table 1:** Demographic attributes of the participants.

Variables and categories		Pretest sample (N=46), n (%)	Formal sample (N=399), n (%)
**Sex**			
	Male	29 (63)	198 (49.6)
	Female	17 (37)	201 (50.4)
**Education**			
	Primary school and below	0 (0)	29 (7.3)
	Junior school	3 (6.5)	82 (20.6)
	High school	5 (10.9)	99 (24.8)
	Professional training college	19 (41.3)	95 (23.8)
	Bachelor and above	19 (41.3)	94 (23.6)
**Income (per month; RMB^a^)**			
	<2500	2 (4.3)	74 (18.5)
	2500-5000	14 (30.4)	215 (53.9)
	5000-10,000	26 (56.5)	90 (22.6)
	>10,000	4 (8.7)	20 (5)
**Marital status**			
	Unmarried	0 (0)	3 (0.8)
	Married	43 (93.5)	330 (82.7)
	Divorced	2 (4.4)	24 (6)
	Widowed	1 (2.2)	42 (10.5)
**Residence status**			
	Living with family	41 (89.1)	319 (79.9)
	Living without family	5 (10.9)	80 (20.1)
**Number of chronic diseases**			
	One or more	19 (41.3)	177 (44.4)
	None	27 (48.7)	222 (55.6)

^a^RMB: Renminbi, the official currency of China; RMB ¥1=US $0.14.

### Ethical Considerations

The study received ethics approval from the University of Southern California-Shanghai Jiao Tong University Institute of Cultural and Creative Industry Ethics Board. The institutional policy does not require the College to issue a separate approval document with a reference number for this type of humanities and social sciences research currently. Participants were provided with a written description of the study before completing the web-based questionnaire, ensuring the confidentiality of their private information solely for the purpose of this study. The completion of the questionnaire indicated approved consent from each participant.

### Measures

#### Social Capital

The measurement of social capital in this study encompassed 2 dimensions: cognitive social capital (trust and reciprocity) and structural social capital (social network and social participation). Given that the study focused on Chinese older adults, the social capital measures were adapted from localized studies. Cognitive social capital was measured by the degree of trust in neighborhood or village councils. In addition, the willingness to engage in actions that benefit others (strangers, neighbors, friends, family, and relatives) was measured (“To what extent would you like to do things that are not directly beneficial to you but are beneficial to others?”) [62]. Structural social capital was assessed by inquiring respondents about the number of interactions with neighbors, relatives, and friends on a 5-point scale (1=1, 2=2, 3=3 to 4, 4=5 to 8, and 5=more than 9) as well as the frequency of engaging in social activities and participating in community events on a scale of 1 (never) to 5 (always) originally [62,63]. However, in the small-scale pretest, the questions on the number of interactions the participants had with their relatives and friends were deleted owing to a factor loading of <0.3.

#### Protective Behavior Intention

The intention to adopt protective behavior measures was drawn upon items derived from the public prevention guidelines of the National Health Commission and the Centers for Disease Control and Prevention [64], as well as the measurement of COVID-19 protective behaviors used in the relevant literature [65]. Respondents were asked to indicate the extent to which they were willing to adopt behaviors related to COVID-19 protection (reducing going out and gathering, wearing a mask, washing hands frequently, and disinfecting promptly). A 5-point Likert scale was used to measure their responses, with options ranging from 1 to 5 (1=hardly, 2=relatively not, 3=neutral, 4=more, and 5=very).

#### Media Exposure

Media types were classified as newspaper and radio, television, and cell phone. Participants were asked to report the amount of time they spent using each type of media per day on a 5-point scale (1=0 minutes, 2=below 1.5 hours, 3=1.5 to 3 hours, 4=3 to 5 hours, and 5=more than 5 hours). In addition, 2 items assessed their attention to COVID-19 messages on the media [66] on a scale of 1 (almost not) to 5 (always) and their self-rated media reliance [67] using a 5-point Likert scale (1=not at all, 2=relatively not, 3=neutral, 4=more, and 5=very).

#### Negative Emotions

Negative emotions in this study were operationalized as 6 distinct emotions: anger, sadness, fear, frustration, helplessness, and worry [21]. Using a 5-point Likert scale ranging from 1 (hardly any) to 5 (very), participants were asked to rate the extent to which they experienced these emotions.

#### Characteristics of the Participants

In this study, we collected demographic information from the participants, including sex, age, education level, income level, marital status, residence status, and chronic disease status. Following independent variables 2-tailed *t* test and one-way ANOVA in SPSS (version 26; IBM Corp), we identified 3 significant variables that had a notable impact on protective behavior intention, namely gender (*P*<.001), educational level (*P*=.047), and income level (*P*=.002). These variables were used as control variables in the subsequent analyses.

### Analysis

In this study, SPSS and MPLUS (version 8.3; Muthén & Muthén) were used to test the reliability of the measurement models. Construct reliability was evaluated based on certain conditions, including a Cronbach α value of >.7 and a composite reliability value >0.7 [68,69]. Average variance extracted is considered to be >0.5 [69], but we can also accept 0.4 [70-73]. Then, a confirmatory factor analysis was conducted to test the model fit. The following fit indices, as suggested by Browne and Cudeck [74], were considered for a reasonably fitting model: a root-mean-square error of approximation of <0.08, Tucker-Lewis index and comparative fit index of >0.9, and ratio of chi-square to *df* (*χ*^2^/*df*) of <5.

To test the research hypotheses, a linear regression analysis was performed. Before conducting the regression analysis, a validated one-way analysis was performed in MPLUS to assess the presence of common method bias. In addition, multicollinearity was evaluated using variance inflation factors, and variance inflation factor values of <10 were considered acceptable, indicating that all predictor variables were available for a multivariate analysis [69]. This analysis was conducted in SPSS. Next, this study tested the mediating and moderating effects using models 4 and 7 of the SPSS macro. Indirect effects were calculated using a bootstrapping technique [75], which involved generating a 95% bias-corrected CI (BC) for indirect effects based on random samples of data. If the BC did not include 0, mediation could be inferred.

## Results

### Means, SDs, and Pearson *r* Correlations

Means, SDs, and Pearson *r* correlations between the main variables are reported in Table 2. It can be seen that the mean value of cognitive social capital was much higher than that of structural social capital. Moreover, the mean value of cell phone exposure exceeded that of the 2 traditional media exposures. Notably, older adults demonstrated a strong overall intention to adopt protective behavior, with negative emotions at a moderate level. In addition, cognitive social capital was significantly correlated with protective intention (*P*<.01), whereas there was a differential correlation between various types of media exposure and the 2 forms of social capital and between various types of media exposure and protective intention.

**Table 2:** Means, SDs, and Pearson *r* correlations of the main variables.

Variables		1. Cognitive social capital	2. Structural social capital	3. Protective behavior intention	4. Newspaper and radio exposure	5. Television exposure	6. Cell phone exposure	7. Negative emotions
**1. Cognitive social capital (mean 4.00, SD 0.56)**								
	*r*	1.000	0.17	0.37	0.07	0.16	0.28	0.002
	*P* value	—^a^	.001	<.001	.14	.002	<.001	.97
**2. Structural social capital (mean 2.71, SD 0.73)**								
	*r*	0.17	1.000	0.06	0.39	0.24	0.02	0.16
	*P* value	.001	—	.21	<.001	<.001	.76	.001
**3. Protective behavior intention (mean 3.97, SD 0.78)**								
	*r*	0.37	0.06	1.000	0.19	0.21	0.23	0.05
	*P* value	<.001	.21	—	<.001	<.001	<.001	.37
**4. Newspaper and radio exposure (mean 2.40, SD 0.78)**								
	*r*	0.07	0.39	0.19	1.000	0.41	–0.06	0.16
	*P* value	.14	<.001	<.001	—	<.001	.90	.002
**5. Television exposure (mean 2.98, SD 0.84)**								
	*r*	0.16	0.24	0.21	0.41	1.000	0.05	0.11
	*P* value	.002	<.001	<.001	<.001	—	.31	.02
**6. Cell phone exposure (mean 3.59, SD 0.75)**								
	*r*	0.28	0.02	0.23	–0.06	0.05	1.000	0.15
	*P* value	<.001	.76	<.001	.90	.31	—	.003
**7. Negative emotions (mean 2.35, SD 0.87)**								
	*r*	0.002	0.16	0.05	0.16	0.11	0.15	1.000
	*P* value	.97	.001	.37	.002	.02	.003	—

^a^Not available.

### Reliability and Validity

**Figure 1:**
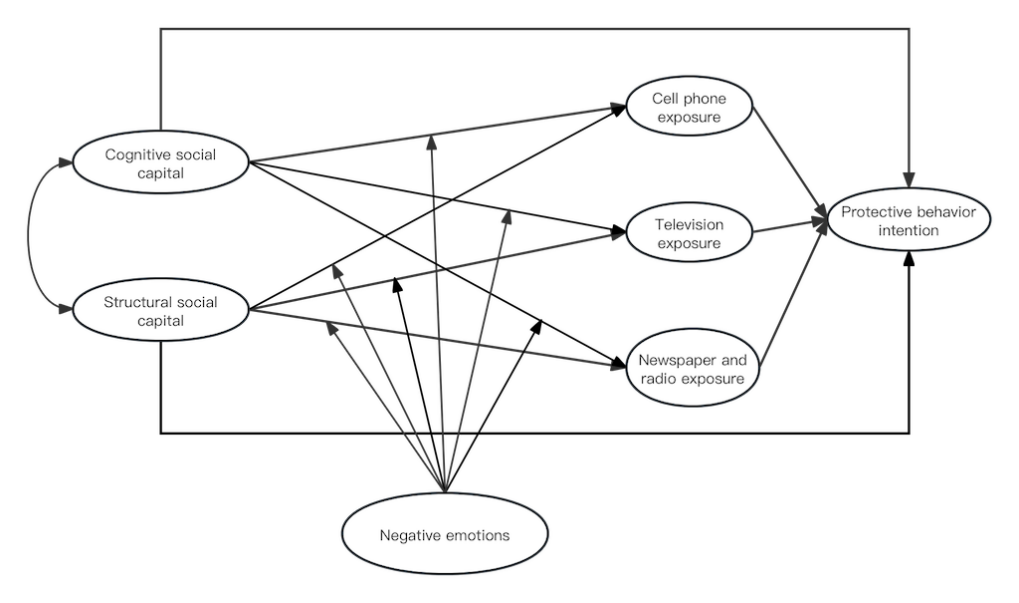
Structural equation model for impacting factors of protective behavior intention among older adults.

In this study, a model consisting of 7 factors was developed (Figure 1). The construct reliability and validity of the model were evaluated based on the following criteria. As shown in Table 3, all the scales’ Cronbach α value and composite reliability value were >.7, indicating good construct reliability. In addition, the average variance extracted was >0.4, suggesting acceptable construct validity. Furthermore, the fit indices of the 7-factor model, which included all the variables in the study, were within the acceptable range after Modification Index correction (*χ*^2^/*df*=2.230, root-mean-square error of approximation=0.056, comparative fit index=0.912, and Tucker-Lewis index=0.901), indicating a good model fit and further supporting the validity of the measurement model.

**Table 3:** The scale’s construct reliability and validity.

Variables	Items (N=33), n (%)	Cronbach α	CR^a^	AVE^b^
Cognitive social capital	6 (18)	.80	0.82	0.46
Structural social capital	5 (15)	.73	0.74	0.41
Newspaper and radio exposure	6 (18)	.83	0.84	0.48
Television exposure	3 (9)	.70	0.71	0.46
Cell phone exposure	3 (9)	.70	0.71	0.46
Protective behavior intention	4 (12)	.84	0.87	0.63
Negative emotions	6 (18)	.92	0.92	0.65

^a^CR: composite reliability.

^b^AVE: average variance extracted.

### Relationship Between Social Capital and Media Exposure

First, this study examined the relationship between social capital and media exposure (Table 4). Controlling for gender, education, and income, older adults with higher levels of cognitive social capital were more likely to use television (B=0.24; *P*<.001) and cell phone (B=0.36; *P*<.001) but not newspaper and radio (B=0.10; *P*=.16). Structural social capital showed a significant positive association with newspaper and radio exposure (B=0.41; *P*<.001) and television exposure (B=0.28; *P*<.001). However, there was no statistically significant association between structural social capital and cell phone exposure (B=0.01; *P*=.92).

**Table 4:** Summary of relationships between social capital and media exposure (N=399).

	Newspaper and radio exposure						Television exposure						Cell phone exposure				
	Model 1^a^			Model 2^b^			Model 3^c^			Model 4^d^			Model 5^e^			Model 6^f^	
	B	*P* value	B		*P* value	B		*P* value	B		*P* value	B		*P* value	B		*P* value
Sex	0.09	.25	0.09		.24	0.07		.43	0.08		.36	0.15		.04	0.18		.02
Education	–0.02	.59	–0.04		.36	–0.07		.12	–0.07		.08	0.14		<.001	0.14		<.001
Revenue	0.004	.95	0.02		.82	–0.03		.70	–0.02		.82	–0.13		.04	–0.11		.08
Cognitive social capital	0.10	.16	—^g^		—	0.24		.001	—		—	0.36		<.001	—		—
Structural social capital	—	—	0.41		<.001	—		—	0.28		<.001	—		—	0.01		.92

^a^*R*^2^=0.01; *F*_4,394_=1.06; *P* value for the *F* test>.05.

^b^*R*^2^=0.15; *F*_4,394_=17.96; *P* value for the *F* test<.001.

^c^*R*^2^=0.04; *F*_4,394_=4.35; *P* value for the *F* test<.01.

^d^*R*^2^=0.08; *F*_4,394_=8.08; *P* value for the *F* test<.001.

^e^*R*^2^=0.12; *F*_4,394_=13.72; *P* value for the *F* test<.001.

^f^*R*^2^=0.05; *F*_4,394_=5.13; *P* value for the *F* test<.001.

^g^The values are not available.

### Relationship Between Social Capital and Protective Behavior Intention

Second, the effect of social capital on protective behavior intention was assessed. The results are presented in Table 5. Cognitive social capital positively predicted protective behavior intention (B=0.52; *P*<.001; model 7), so hypothesis 1a was supported. However, there was no significant association between structural social capital and protective behavior intention (B=0.07; *P*=.21; model 8), so hypothesis 1b was not supported.

**Table 5:** Summary of regressions predicting protective behavior intention (N=399).

	Model 7^a^			Model 8^b^			Model 9^c^			Model 10^d^	
	B	*P* value	B		*P* value	B		*P* value	B		*P* value
Sex	0.20	.007	0.24		.003	0.17		.03	0.18		.02
Education	–0.01	.85	–0.004		.92	–0.02		.62	–0.02		.56
Revenue	–0.12	.06	–0.10		.14	–0.10		.12	–0.07		.25
Cognitive social capital	0.52	<.001	—^e^		—	0.43		<.001	—		—
Structural social capital	—	—	0.07		.21	—		—	–0.03		.63
Newspaper and radio exposure	—	—	—		—	0.13		.01	0.14		.01
Television exposure	—	—	—		—	0.08		.08	0.12		.01
Cell phone exposure	—	—	—		—	0.14		.005	0.23		<.001

^a^*R*^2^=0.18; *F*_4,394_=21.34; *P* value for the *F* test<.001.

^b^*R*^2^=0.05; *F*_4,394_ =4.91; *P* value for the *F* test<.001.

^c^*R*^2^=0.22; *F*_7,391_=16.13; *P* value for the *F* test<.001.

^d^*R*^2^=0.14; *F*_7,391_ =9.03; *P* value for the *F* test<.001.

^e^The values are not available.

### Mediating Effects of Media Exposure

Next, this study evaluated the mediating effects of 3 types of media exposure between social capital and protective behavior intention (Table 5). According to model 9, the positive effect of cognitive social capital on protective behavior intention remained significant after the mediating variable cell phone exposure was added (B=0.43, *P*<.001). By contrast, after the mediating variables were added to the regression model of structural social capital and protective behavior intention, newspaper and radio exposure showed a positive effect on protective behavior intention (B=0.14; *P*=.01), television exposure (B=0.12; *P*=.01), and cell phone exposure (B=0.23; *P*<.001; model 10).

Bootstrap repeated sampling analysis (Table 6) examined the indirect effects and provided further insights into the mediating effect of media exposure between social capital and protective behavior intention. Mediated by cell phone exposure, the effect value of cognitive social capital on protective behavior intention was 0.05, with a 95% BC of 0.01-0.11, which does not contain 0, so hypothesis 2c was verified. By contrast, when newspaper and radio exposure acted as a mediator, the indirect effect value was 0.01, with a 95% BC of –0.003 to 0.04, so hypothesis 2a was not supported. Similarly, television exposure was not confirmed as a mediating variable between cognitive social capital and protective behavior intention, so hypothesis 2b was rejected.

**Table 6:** Direct and indirect effects of social capital on protective behavior intention via media exposure.

Path	Direct effect		Indirect effect	
	Effect size (SE)	95% BC^a^	Effect size (SE)	95% BC
1^b^	0.43 (0.06)	0.30 to 0.57 ^c^	0.01 (0.01)	–0.003 to 0.04
2^d^	0.43 (0.06)	0.30 to 0.57	0.02 (0.01)	–0.004 to 0.05
3^e^	0.43 (0.06)	0.30 to 0.57	0.05 (0.03)	0.01 to 0.11^c^
4^f^	–0.03 (0.06)	–0.14 to 0.08	0.06 (0.02)	0.01 to 0.11^c^
5^g^	–0.03 (0.06)	–0.14 to 0.08	0.03 (0.02)	0.005 to 0.07^c^
6^h^	–0.03 (0.06)	–0.14 to 0.08	0.001 (0.01)	–0.03 to 0.03

^a^BC: bias-corrected and accelerated CI.

^b^When newspaper and radio exposure acts as a mediating variable between cognitive social capital and protective behavior intention.

^c^Mediation is assumed because bias-corrected and accelerated CI does not exceed 0.

^d^When television exposure acts as a mediating variable between cognitive social capital and protective behavior intention.

^e^When cell phone exposure acts as a mediating variable between cognitive social capital and protective behavior intention.

^f^When newspaper and radio exposure acts as a mediating variable between structural social capital and protective behavior intention.

^g^When television exposure acts as a mediating variable between structural social capital and protective behavior intention.

^h^When cell phone exposure acts as a mediating variable between structural social capital and protective behavior intention.

In addition, when newspaper and radio exposure and television exposure were seen as mediating variables for the effect of structural social capital on protective behavior intention, the upper and lower limits of bootstrap 95% CIs did not contain 0, indicating that structural social capital was significantly associated with protective behavior intention through both mediating paths of newspaper and radio exposure and television exposure; thus, hypotheses 2d and 2e were supported. By contrast, cell phone exposure was not proved as a mediator between structural social capital and protective behavior intention, so hypothesis 2f was not supported.

### Moderating Effect of Negative Emotions

**Figure 2:**
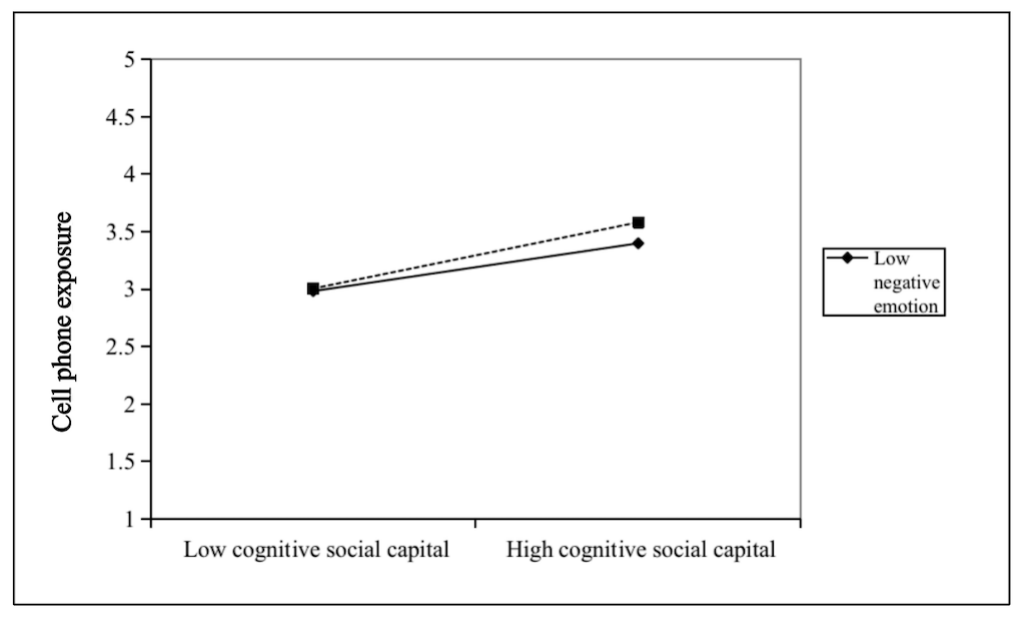
Moderating effect of negative emotions between cognitive social capital and cell phone exposure.

Finally, this study tested the moderating effect of negative emotions between social capital and media exposure (Table 7). According to model 11, the interaction term between cognitive social capital and negative emotions had a significant positive effect on cell phone exposure (B=0.14; *P*=.03). However, according to models 12 and 13, the interaction term of structural social capital and negative emotions was not statistically significant for newspaper and radio exposure or television exposure. A further simple slope analysis of the moderating effect of negative emotions between social capital and media exposure (Figure 2) showed that, on the one hand, for research participants with higher levels of negative emotions (Mean+1SD), cognitive social capital had a significant positive predictive effect on cell phone exposure (simple slope=0.52; t_395_=4.36; *P*<.001). On the other hand, for participants with lower levels of negative emotions, cognitive social capital positively influenced cell phone exposure to a lesser extent (simple slope=0.37; t_395_=5.95; *P*<.001). In addition, the mediating effect of cell phone exposure on the relationship between cognitive social capital and protective behavior intention tended to increase at all 3 levels of negative emotions. Thus, cognitive social capital was more likely to enhance older adults’ protective behavior intention by increasing their cell phone exposure as older adults’ levels of negative emotions rose. Therefore, hypothesis 3 was partially supported.

**Table 7:** The moderating effect of negative emotions between social capital and media exposure.

	Cell phone exposure		Newspaper and radio exposure		Television exposure	
	Model 11^a^		Model 12^b^		Model 13^c^	
	B	*P* value	B	*P* value	B	*P* value
Sex	0.13	.07	0.08	.28	0.07	.41
Education	0.14	<.001	–0.04	.30	–0.08	.06
Revenue	–0.12	.05	0.02	.72	–0.01	.91
Cognitive social capital	0.37	<.001	—^d^	—	—	—
Structural social capital	—	—	0.40	<.001	0.27	<.001
Negative emotions	0.10	.01	0.09	.04	0.07	.12
int1^e^	0.14	.03	—	—	—	—
int2^f^	—	—	0.002	.97	–0.02	.81

^a^*R*^2^=0.39; *F*_6,392_=11.45; *P* value for the *F* test <.001.

^b^*R*^2^=0.16; *F*_6,392_=12.74; *P* value for the *F* test <.001.

^c^*R*^2^=0.29; *F*_6,392_=5.82; *P* value for the *F* test <.001.

^d^The values are not available.

^e^Interaction items of cognitive social capital and negative emotions.

^f^Interaction items of structural social capital and negative emotions.

**Figure 3:**
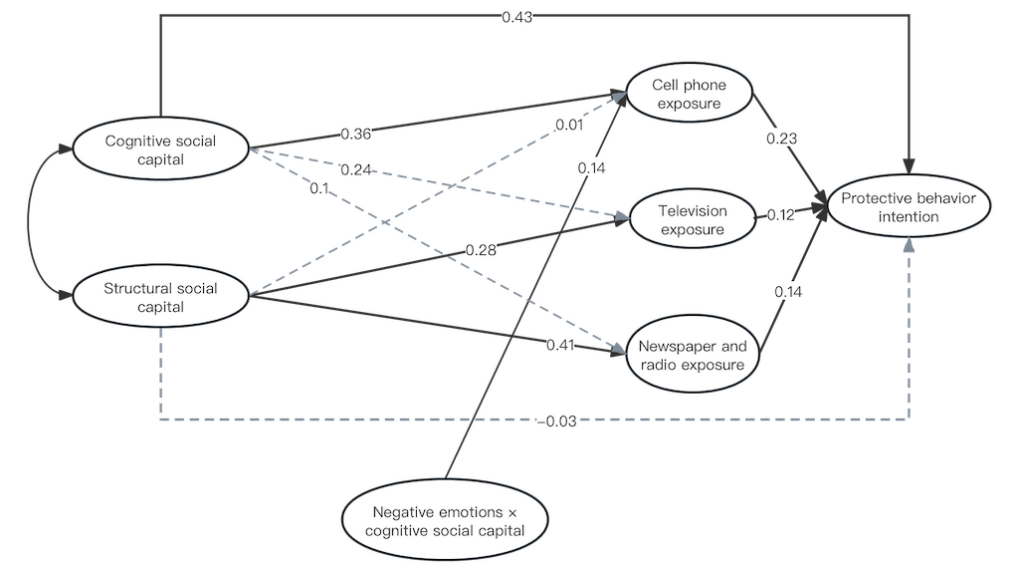
Structural equation model and effect values for impacting factors of protective behavior intention among older adults.

Figure 3 illustrates the correlations and effect values among the variables used in this study. Notably, cognitive social capital exhibited a significant correlation with protective behavior intention (*P*<.001), with the mediating effect of cell phone exposure established between the 2 variables (*P*<.001). In addition, the mediating effect of newspaper and radio exposure and television exposure between structural social capital and protective behavior intention was established (*P*<.001), whereas negative emotions played a moderating role in the relationship between cognitive social capital and protective behavior intention (*P*<.001).

## Discussion

### Principal Findings

This study aimed to investigate the applicability of the SIM in the social context of the post–COVID-19 epidemic era in China. The study focused on assessing the reliability of social capital in influencing health protection intentions through media exposure, with a specific emphasis on the older Chinese population. This study categorized social capital into cognitive and structural dimensions and examined media exposure from the perspective of 3 main media types: newspaper and radio, television, and cell phone. Considering the risk characteristics of COVID-19 messages and the influence of selective exposure, this study also tested whether negative emotions play a moderating role between social capital and media exposure.

The results of this study support the SIM by indicating that potential factors at the personal, interpersonal, and social levels can explain individuals’ intention to adopt health behaviors. First, cognitive social capital was shown to promote health behavior intention, whereas structural social capital did not have a significant effect on health behavior intention, which is consistent with the findings of De Silva et al [40] on social capital and mental health outcomes. However, the underlying reasons for these differences were not fully elucidated and should continue to be explored in depth in future research.

Second, the mediating role of media exposure between social capital and health behavior intention was also emphasized, validating the SIM pathway. Interestingly, the results revealed that the 2 types of social capital influenced protective behavior intention through different patterns of media exposure. Cognitive social capital positively influenced cell phone exposure, indicating that older adults with higher levels of trust and reciprocity were more inclined to use new media for health information seeking and adopt protective behavior. By contrast, structural social capital predicted health behavior intention through newspaper and radio exposure and television exposure, with a stronger mediating effect observed for newspaper and radio exposure. This implies that older adults with more social engagement and larger social networks prefer traditional media, engaging in more offline information activities. Previous studies have suggested that people tend to communicate information across all (or many) media channels [76]. Therefore, most studies have not categorized media channels when examining the relationship between social capital and health information seeking. However, according to the findings of this study, it may be advantageous for health communicators to target different characteristics of the population using different media channels when disseminating messages rather than solely focusing on increasing people’s social capital through welfare measures.

In addition to the association among social capital, media exposure, and protective behavior intention, the results of this study illustrate the importance of negative emotions. The higher the level of negative emotions, the more pronounced the positive effect of cognitive social capital on cell phone exposure, validating the findings of previous studies that negative emotions have a positive effect on information seeking [77,78]. By contrast, the results of this study indicate that negative emotions did not cause interference during the influence of traditional media exposure, such as newspaper and radio exposure and television exposure, among older adults. As numerous studies have pointed out, the intensity of negative emotions during pandemics interacts with the impact of efficiently disseminated media [79,80]. Consistent with this, this study also suggests that new media exhibit a more pronounced correlation with negative emotions than traditional media. However, the specific direction of influence between negative emotions and media exposure remains unclarified in this study. Although some studies indicate that negative emotions may prompt individuals to seek information from new media [52,81,82], a substantial body of evidence also suggests that an overload of information can significantly induce panic, anxiety, and frustration [83-85].

Although the threshold of emotion varies from person to person [86] and it is difficult to define an optimal level that can be generalized, future research should explore the differences in the effects of negative emotions in specific social and media environments, as well as the reasons that trigger such differences. As a result, public sentiment can be better appeased during health crises, thus encouraging healthy behavior. Furthermore, the findings also suggest the need to explore other factors influencing health communication and behavior to extend the SIM to improve its applicability in relevant research.

### Limitations

First, this study used an web-based questionnaire to recruit the sample, which may have introduced a bias in the sample. It is possible that a significant portion of the sample had access to and preferred using the internet for information seeking. Although efforts were made to involve relatives in completing the questionnaire on behalf of older adults who did not use the internet, the effectiveness of this approach in capturing the perspectives of non–internet users could not be fully controlled or measured. Thus, the generalizability of this study may be limited to this sampling method.

Second, the cross-sectional nature of this study, in which participants’ responses were recorded at a single point in time, restricted the ability to draw causal conclusions.

The relationships observed between the variables may be subject to reverse causality or the influence of unmeasured confounding variables. However, the SIM assumes a directional relationship among social factors, information behavior, and health outcomes [12], and this study did demonstrate a pattern of association in this direction. Future research can use longitudinal designs or experimental approaches to better understand the temporal and causal dynamics among social capital, media exposure, negative emotions, and health behavior intention.

Third, this study attempted to include the element of negative affect to extend the SIM. In fact, there may be additional variables that may influence information behavior and health outcomes. Future research should consider incorporating additional relevant factors to enhance the comprehensiveness and applicability of the SIM.

### Conclusions

This study partially supports the SIM by indicating that social capital can directly influence health-protective behavior intention. In addition, the results suggest that media exposure can act as an additional bridge in this pathway of influence and that negative emotions can contribute to increased older adults’ engagement in epidemic information communication via cell phones. Therefore, in the context of the post–COVID-19 epidemic era, health communicators need to focus not only on message delivery but also on developing targeted health communication strategies. Consequently, communication effectiveness could be enhanced for older adults who are in health vulnerability as well as information vulnerability. Future research should continue to explore theories and applications of the SIM to discover more intervention points to promote universal health behaviors.

## Abbreviations

BC: bias-corrected CI
SIM: structural influence model of communication

## Data Availability

The data sets generated and analyzed during this study are not publicly available to protect the privacy of participants but are available from the corresponding author upon reasonable request.
